# The INSCHOOL project: showcasing participatory qualitative methods derived from patient and public involvement and engagement (PPIE) work with young people with long-term health conditions

**DOI:** 10.1186/s40900-023-00496-5

**Published:** 2023-10-12

**Authors:** Bethan Spencer, Siobhan Hugh-Jones, David Cottrell, Simon Pini

**Affiliations:** 1 University of Leeds, Leeds Institute of Health Sciences, Leeds, UK; 2 School of Psychology, University of Leeds, Leeds, UK

**Keywords:** Patient and public involvement and engagement, Participatory methods, Qualitative methods, Creative methods, Young people, Children

## Abstract

**Background:**

Evidence suggests resources and services benefit from being developed in collaboration with the young people they aim to support. Despite this, patient and public involvement and engagement (PPIE) with young people is often tokenistic, limited in engagement and not developmentally tailored to young people. Our paper aims to build knowledge and practice for meaningfully engaging with young people in research design, analysis and as research participants.

**Methods:**

We report the participatory processes from the INSCHOOL project, examining long-term health conditions and schooling among 11–18 year olds. Young people were consulted at the inception of the project through a hospital-based youth forum. This began a partnership where young people co-designed study documents, informed the recruitment process, developed creative approaches to data collection, participated in pilot interviews, co-analysed the qualitative data and co-presented results.

**Results:**

PPIE advisors, participants and researchers all benefitted from consistent involvement of young people throughout the project. Long-term engagement allowed advisors and researchers to build rapport and facilitated openness in sharing perspectives. PPIE advisors valued being able to shape the initial aims and language of the research questions, and contribute to every subsequent stage of the project. Advisors co-designed flexible data collection methods for the qualitative project that provided participants with choices in how they took part (interviews, focus groups, written tasks). Further choice was offered through co-designed preparation activities where participants completed one of four creative activities prior to the interview. Participants were therefore able to have control over how they participated and how they described their school experiences. Through participatory analysis meetings advisors used their first-hand experiences to inform the creation of themes and the language used to describe these themes. PPIE in every stage of the process helped researchers to keep the results grounded in young people’s experience and challenge their assumptions as adults.

**Conclusions:**

Young people have much to offer and the INSCHOOL project has shown that researchers can meaningfully involve young people in all aspects of research. Consistent PPIE resulted in a project where the voices of young people were prioritised throughout and power imbalances were reduced, leading to meaningful participant-centred data.

## Abstract (plain language summary)

**Background:**

Young people’s voices are often overlooked in research about long-term health conditions, but evidence suggests involving young people improves research quality, usefulness and impact. Despite the benefits, young people are not involved in research as often or as well as they could be.

**Methods:**

Young people were consulted during the early planning of the INSCHOOL project and began a long-term co-production partnership. Through a series of meetings, young advisors helped to design project methods, took part in pilot interviews, analysed interview data, and presented results. Co-designed activities helped participants prepare for their interviews, which was a key part in helping participants tell their own stories.

**Results:**

Having young advisors throughout the research added significant value to the project design and helped the results remain grounded in young people’s experiences. The co-designed methods gave participants time to prepare for interviews, which allowed them to feel more comfortable and in control of the interview process. This reduced the amount of influence the researchers had over the content of the interviews and gave young people an opportunity to describe experiences of school life that were meaningful to them. PPIE advisors, participants and researchers all benefitted from consistent involvement of young people throughout the project. Long-term engagement allowed advisors and researchers to build rapport and facilitated openness in sharing perspectives.

**Conclusions:**

Researchers should aim to engage with young people at an early stage of research to develop partnerships that allow them to shape the direction and content of the research, and how the information is analysed and presented. Co-designing methods that offer flexibility and make it easy for young people to have more control over the research process can lead to more meaningful and valuable results.

## Background

Young people’s voices are often overlooked in research about chronic health conditions, but evidence suggests resources and services benefit from being developed in collaboration with the young people they aim to support [1–3]. Facilitating the voices of young people is an important and evolving discourse within childhood research, with debate around claims of “authenticity” and the relative value of voices removed from their context [4, 5]. There is a lack of consistency in the terminology in this area of research [6, 7], with many “blurred boundaries” between qualitative research, patient and public involvement, user testing, and co-production/co-design [7]. There is call for greater “authentic collaboration” [8] between disciplines in this area [4, 7] with researchers selecting approaches based on methodological and pragmatic considerations [7]. This article reports an example of a pragmatically driven approach to engaging with young people in the roles of advisors, co-designers and research participants. “Patient and public involvement and engagement (PPIE)” is the term used to represent any work we have conducted where we engaged with young people in our research process outside of their role as research participants in the qualitative study.

Including the lived experiences of young people in the design and conduct of research has proven beneficial for the quality and applicability of healthcare research and service development [2, 3, 9, 10]. Researchers are encouraged to adopt a “no research about me without me” [11] approach to designing research “with” young people [4]. PPIE is not only about “doing the right thing” for researchers, but provides another form of knowledge and challenge to existing knowledge, which keeps researchers accountable to the realities of people’s lives. There are well documented benefits when PPIE is meaningfully included in the research process, which include empowerment, confidence, research skills, sharing with others in similar positions, building of rapport with researchers and meaningfully contributing to research knowledge and future care [10, 12–14]. However, despite the benefits of involving young people throughout the research process, PPIE is rarely used at all stages of research, often limited in its level of engagement [15–18] and can be prone to “tokenism” [7]. There is a continuum of PPIE ranging from minimal levels of engagement through to fully egalitarian research partnerships [19]. Previous research that has engaged with young people has been described as predominantly “involving” and “consulting” with them, but less commonly facilitating participation directly in the research process through allowing young people to “lead” or actively “support” research and knowledge generation [6].

Early direct engagement with PPIE advisors is recommended to create a good working environment, share the research agenda with those it aims to support, and to set the direction of the collaboration throughout the research process [13, 20, 21]. However, effective PPIE takes time and thoughtful planning, and no single approach will be appropriate for every young person [22]. A lack of time and funding can make it difficult for researchers to develop meaningful ways to incorporate PPIE with young people into their research process [1, 6, 10, 12, 18, 22–24]. PPIE advisors can also feel overwhelmed by the research tasks, especially when there is not adequate preparation for them or when PPIE activities are not tailored to their level of ability and experience [10, 21]. These barriers compromise the potential significant benefits that research and services can gain from PPIE with young people, but can be mitigated to some extent through provision of training and mentoring [14].

Participatory research is a collective term for the development and application of research designs, methods, and frameworks in direct collaboration with those affected by the issue being studied [25, 26]. Within participatory research there are a variety of interrelated research approaches, including participatory evaluation, collaborative research and participatory action research [27–30]. Participatory research can be seen as an extension of the philosophy of PPIE into the methodology of research projects that look to meaningfully include the voices of key stakeholders. The use of participatory methods in child health research has increased in recent times, but has been slow to develop in marginalised or vulnerable groups of young people [31]. A wide diversity of frameworks for participatory research have emerged to represent specific populations, research questions, methods and aims [26]. As well as common engagement methods, such as interviews, focus groups and youth panels [2, 6], using engaging participatory approaches to prioritise young people wherever possible within research has been shown to be valuable in generating discussion and meaningful participation [1, 2, 16, 17, 32, 33]. Examples of this range from broad approaches such as engaging directly with local community activities [21] and social events [34], through to more specific methods including photo-elicitation [35], modelling [36] and card-sorting activities [33]. Despite a range of possible creative participatory methods being available, having a “toolbox” approach to offer flexibility in data collection can be preferable to selecting one specific method [37].

A recent review of research with young partners by experience called for more reflection on research partnerships with young people [22]. The INSCHOOL project began in 2021 with the aim of investigating the school lives of young people living with long-term physical health conditions, and for young people to be able to describe their school experiences in their own words with their own priorities. A qualitative evidence synthesis of research assessing the school experiences of young people was conducted [38] before development of a qualitative workstream which aimed to explore lived experience of young people in greater depth. An important additional aim of this research was to facilitate the inclusion of young people’s voices at every stage of the research process. Time, planning and funding for PPIE was factored into the project from the outset of the proposal development. This paper provides a detailed worked example of the approaches used to engage young people in the first two years of the INSCHOOL project through PPIE activities and how this directly informed the participatory methods used in the qualitative workstream.

## PPIE and development of participatory methods

The following sections describe the methods used throughout the INSCHOOL qualitative workstream, with descriptions of the associated PPIE work with young people, and how this influenced design of the methods. Reflections from PPIE advisors and researchers are included throughout. All PPIE work was led by an experienced child health researcher (SP), and a research assistant (BS), with support in organisation and facilitation by youth workers from Leeds Children’s Hospital.

### PPIE groups

During the creation of the INSCHOOL project, the research team partnered with Leeds Youth Forum (LYF), who provided an opportunity for consistent engagement with young people. PPIE work with LYF began in June 2019 and ran until the end of the qualitative project in July 2023. LYF will continue to advise the INSCHOOL project until its completion in August 2026. Facilitated by youth workers from Leeds Children’s Hospital, LYF are a group of young people aged 11–25 with a range of experiences of the healthcare system who meet regularly to discuss health and healthcare. LYF regularly add new members, therefore new young people were consistently able to engage in the PPIE work and offer their perspectives. The INSCHOOL team met with LYF approximately every three months to provide updates on the project and listen to their feedback. Additional meetings were scheduled when there was an opportunity to invite them to be more directly involved in a particular aspect of the project. In these meetings the particular activity was described (e.g. designing study documents, engaging in pilot interviews or participatory analysis) and LYF members could ask questions before deciding if they wished to be a PPIE advisor for that activity.

Young people said they had joined the PPIE work in order to feel like they were making a difference and to have the chance to offer their opinions on issues that have personally impacted them.

“*I wanted to help other people who are going through a similar situation to me and hopefully support the ones in the future by making their life a little bit easier*.”

PPIE advisors found it was helpful when the researchers were concise and kept everyone informed throughout the process. Also, it was important for them to feel listened to, valued and understood.

“*I think they really took on board what I was saying and I think that this is evident in the final outcomes*.”

Any young person who engaged in PPIE activities outside of the scheduled LYF meetings were given a voucher to thank them for their time.

### Inception of the project

At an early stage in the creation of the proposal for the INSCHOOL project, when only the broad topic was outlined, an initial meeting was arranged with LYF to introduce the research agenda. Engagement at this early stage allowed their experiences to guide the research direction and priorities. LYF shared their experiences of school life in the context of their health conditions and highlighted some of the most important aspects of these experiences and the challenges they faced. To inform development of the methods, they were asked to imagine how young people might feel if they were invited to talk about these experiences with a researcher, and what their concerns or questions might be. This began an ongoing dialogue with LYF about appropriate methods and how this would be applied in the INSCHOOL project, which will be described throughout this paper. The meeting concluded by outlining the stages of the project and how they would like be involved on an ongoing basis.

Alongside meeting young people, the inception of the project involved early engagement with clinical collaborators to discuss the school experiences raised in their clinics, what support is currently offered, and the potential utility of this research within their clinical practice.

In the initial meeting PPIE advisors made meaningful contributions to the design of the research question:

“*What impact do long-term health conditions have on the school lives of young people?*”

The term *“impact”* was preferred to alternatives such as *“experiences”* and *“outcomes”* because of the more direct relation to how it felt to live with their conditions and how their lives were affected. *“Long-term”* was preferred to *“chronic”* because of their perceived negative connotations associated with this term. *“Health conditions”* was preferred to *“illness”* for the same reasons. PPIE advisors preferred to be referred to as *“young people”*, rather than *“children”* or *“adolescents/teenagers”*, and *“school lives”* was agreed to capture the holistic aspects of their experiences.

### Recruitment process for research participants in the qualitative project

Clinical collaborators were identified in 11 clinics across Leeds Children’s Hospital and participants were recruited to the qualitative study through these clinicians, with the support of research nurses. Young people were considered eligible if they were between 11 and 18 years, attended mainstream secondary school, and were cared for within one of the 11 clinics: oncology, chronic pain, cystic fibrosis, diabetes, neuromuscular, asthma, rheumatology, allergies, dermatology, colorectal and haematology. They were approached in outpatient clinics by clinical staff with information about the project and the option to fill in a “consent to contact” form to give permission for their details to be shared with the research team.

PPIE advisors emphasised the importance of friendliness and informality when professionals were introducing the project to potential research participants in clinic. Based on that recommendation, young people and their parents who completed a consent-to-contact form were approached informally by telephone and, if unsuccessful in the first instance, followed up by text, and finally via email if contact remained unsuccessful. These phone calls provided an opportunity to talk informally about the project, ask and answer questions, discuss the project with parents if they were under 16, and begin to establish a positive rapport. Contact details for the researchers were given so parents and participants could get in touch at any time with further questions or concerns about the project. Young people were reminded they could decline participation at any stage of this initial consent process.

**Fig. 1:**
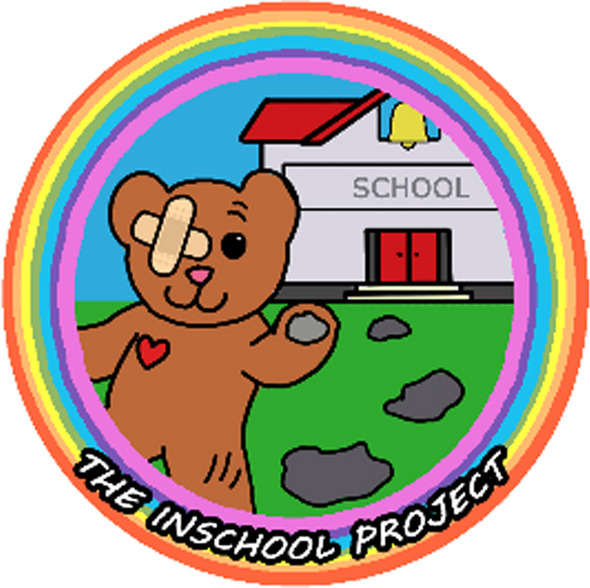
The INSCHOOL Project logo designed by a LYF member

PPIE advisors shaped the language and structure of the recruitment documents by telling us what they would want to know about the project if they were being asked to participate and how they would explain this to other young people. Copies of these documents and links to online consent/demographic forms were sent to any willing participants in a welcome email following initial telephone contact. Hard copies were posted to those who preferred this option. All study documents and emails included the project logo (Fig. 1), which was designed by a member of LYF after a logo design competition was held. These documents included: separate information sheets for 11-15years, 16-18years and parents/carer of 11-15years; a simplified flowchart version of the information sheet accessible for all ages; online consent forms for 16-18years and parents/carers of 11-15years; online assent forms for 11-15years; and online demographic forms to collect personal, health and school information.

At this early stage of the process the project logo competition proved to be an easy and successful way to engage LYF and PPIE advisors in the research topic and build a rapport with the research team. The logo they created is visually appealing and captures the project (school and health), but also their desire for it to represent inclusivity (through the rainbow border).

PPIE sessions highlighted flexibility as a key component of the data collection to ensure potential research participants were not precluded from participation because of logistics or perception of the process. Interviews and focus groups were conducted online via Zoom, and were arranged at any time that suited the participant. Telephone interviews were offered to those without the means to connect easily to Zoom. Participants could also choose whether they would feel more comfortable with a parent or family member present.

Despite the requirements from Health Research Authority and the Research Ethics Committee to include specific wording for information around consent and data management, the language and tone used in study documents and initial approaches to participants in clinics was significantly influenced by the PPIE. They emphasised an informal, friendly, flexible approach, with clear and uncomplicated information about the study, showing the value of PPIE in optimising the reach of research to young people who may typically see barriers to participation.

### Data collection

PPIE advisors highlighted that some research participants may be uncomfortable sharing their stories in a group setting, whereas others might prefer this option. Therefore participants were offered a choice between an interview and a small focus group, and indicated their preference when completing the demographic form. On this form, participants could also suggest a question they would like to be asked first in the interview, which aimed to reduce any anxiety about how the interactions might begin and give them some control over this first interaction. Following completion of the online assent/consent and demographic forms, those selecting an interview were contacted to arrange a time and those selecting a focus group were added to a waiting list until others selected this option. At this stage they were again reminded of their right to withdraw and asked if they had further questions about the project. When enough participants selected a focus group they were informed of the basic demographics of the other participants who would be attending the group, so they could make a more informed decision about whether they were comfortable proceeding having considered age, gender, and health conditions of other group members. During recruitment conversations for focus group participants, they were asked to provide consent for this level of information to be shared with other focus group participants.

Prior to the qualitative project opening to recruitment, four pilot interviews were conducted with PPIE advisors (2 from LYF, 1 from Leeds Research Owls, and 1 from an oncology PPIE group). These pilot interviews gave the PPIE advisors first-hand experience of the research process so they could provide informed feedback on the data collection methods. It also gave researchers an opportunity to test, practice and refine their approach. The pilot interviews were very positive and the PPIE advisors valued the opportunity to actively participate and learn more about the planned research process.

PPIE advisors who participated in the pilot interviews reinforced the appropriateness of the proposed data collection plans and offered important guidance about the language used around chronic health conditions. For example, any reference to “*illness*” was removed from the documents and the language used by the researchers and replaced by “*health condition*”. Two of the pilot interview advisors were subsequently involved in the participatory analysis and could use their first-hand experience of the methods and their increased rapport with the research team to enhance their perspective of the data and engagement in the activities.

**Fig. 2:**
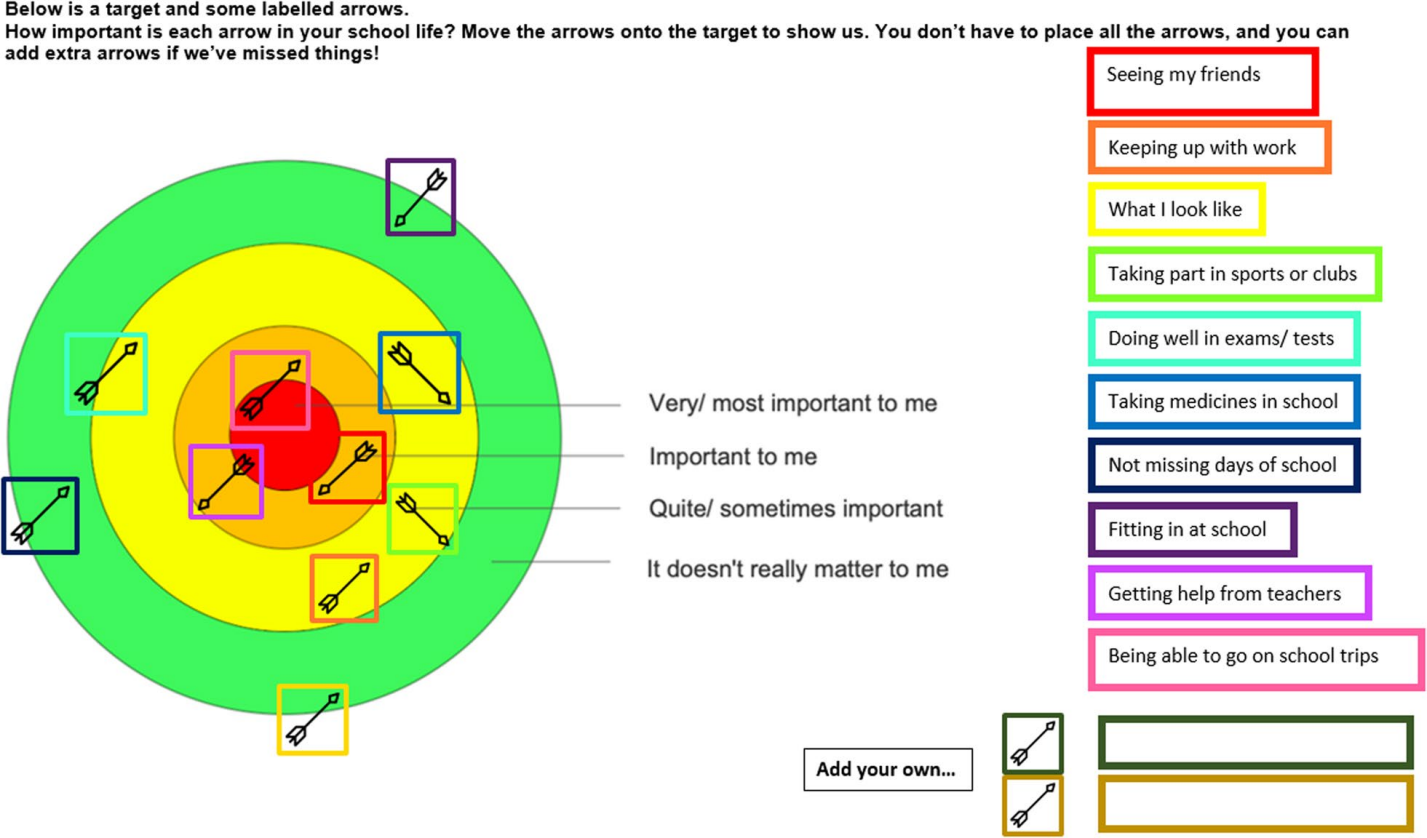
Target activity completed by 13 year-old from the colorectal surgery clinic

**Fig. 3:**
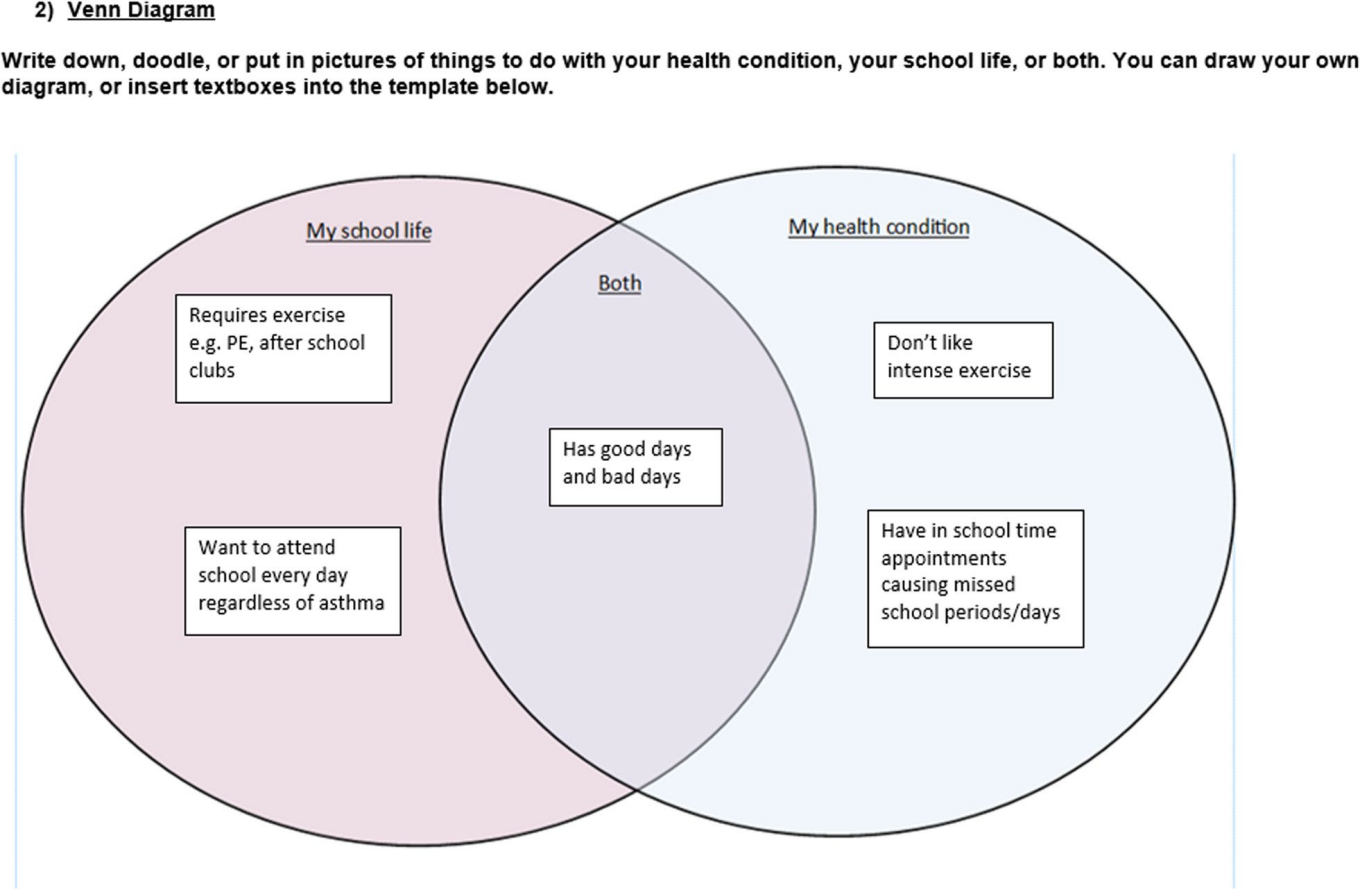
Venn diagram activity completed by 13 year-old from the asthma clinic

**Fig. 4:**
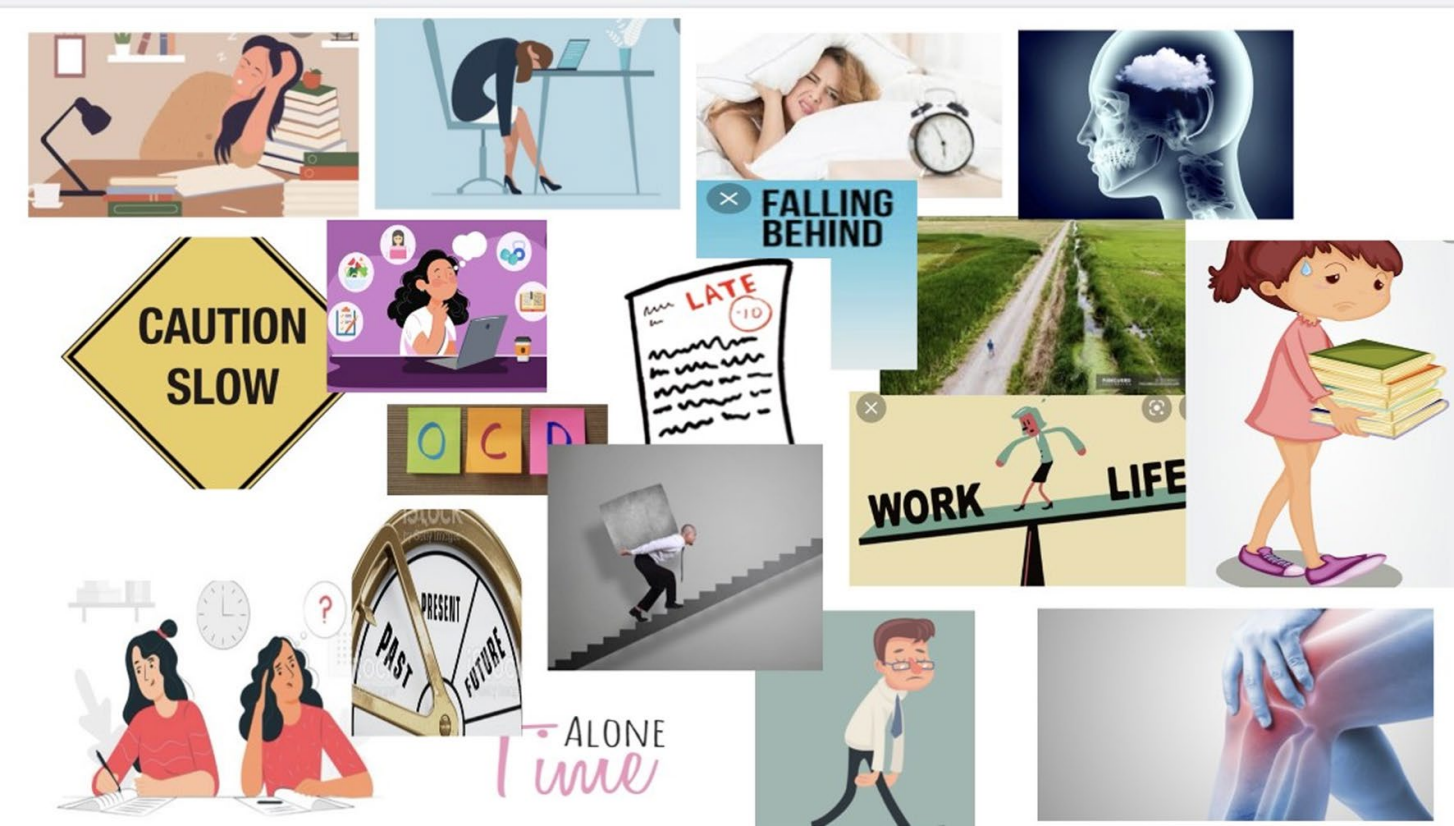
Mood board activity completed by 18 year-old from the rheumatology clinic

**Fig. 5:**
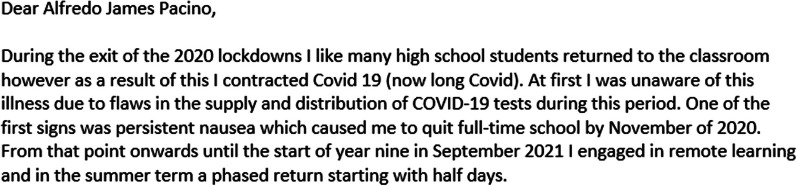
Letter writing activity completed by 14 year-old from the dermatology clinic

When developing the methods we wanted to optimise the voices of our participants by giving them as much time as they needed in the consent process to ensure they felt comfortable in what they were being asked to take part in, and then continuing this process by giving them time to prepare for the interview/focus group. With that in mind, once we had established informed consent, young people were given the option to complete a short creative preparation activity before the interview or focus group. The aim of these activities was to give participants time and a framework in which to think about what they might want to say in order to help them feel more comfortable and confident in telling their own stories. We believed this was essential to generate rich, thoughtful and relevant interview data in a way that felt more under the control of young people, i.e. giving them time and ways to think about what aspect of their lived experience they wanted to share, and what to prioritise. These preparation activities were co-designed in a PPIE meeting where advisors engaged with and discussed seven possible activities. The seven initially proposed activities were sketched out by the research team following a discussion in a LYF meeting where young people expressed a general preference for visually engaging activities or activities that were quick, easy and practical. The PPIE advisors selected four of the proposed activities to develop, which they felt would appeal to young people with a range of interests. They developed the wording and visual design of the activities, discussed how these activities could be presented to participants in the qualitative study and what information they would need. Participants in the qualitative study were asked to choose one of these activities to complete and bring with them to their interview. The choice of activities were sent via email and each activity included completion instructions, which were also described in initial telephone contact with the participant. Participants were advised to contact the researcher if they had any questions. Examples of completed versions of the activities can be seen in Figs. 2, 3, 4 and 5. The target activity (Fig. 2), asked participants to prioritise aspects of their school life by dragging the associated arrows into the target. They could choose from labels pre-defined by PPIE advisors and a literature review [38], or create their own. The Venn diagram (Fig. 3) asked them to add key words or phrases for the important aspects of “school” and “health” into the relevant circles and then start to think about where those two parts of their life overlapped. The mood board activity (Fig. 4) asked participants to take their own photos or gather images from other sources (e.g. the internet) to show us how their health condition affected their life at school. The letter writing activity (Fig. 5) asked participants to write a letter to a celebrity of their choice (or any other important person to them) and tell them about what it was like to have their health condition and how it affected their life at school.

**Fig. 6:**
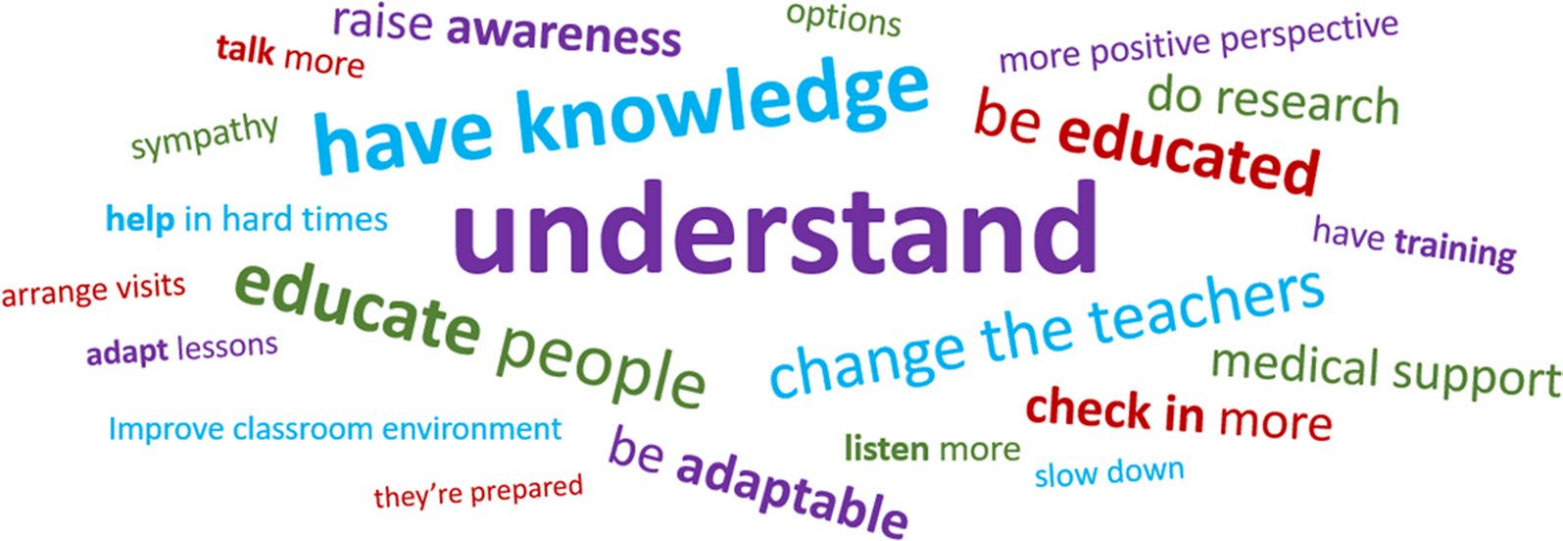
Word cloud advice given for schools/teachers

**Fig. 7:**
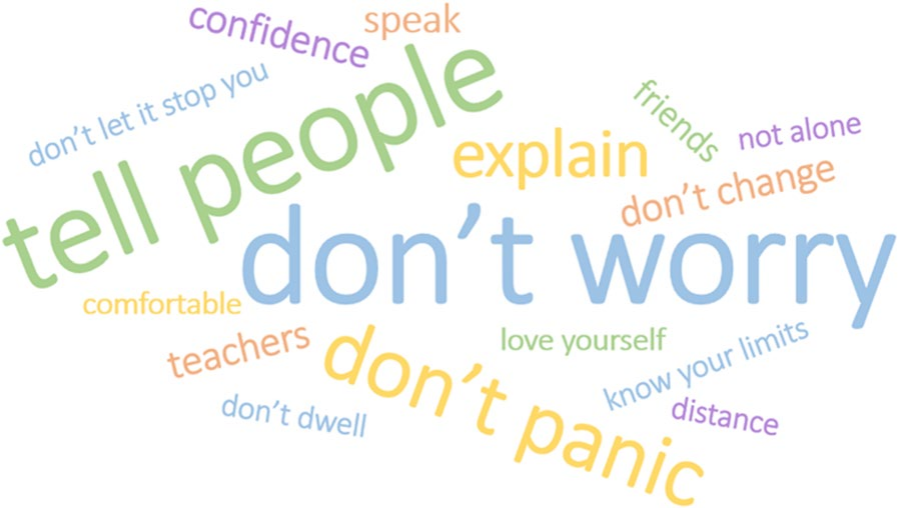
Word cloud of advice from young people to young people

The preparation activities were an important part of facilitating the participant driven aim of the data collection. To open the interviews, after verbally confirming their consent, the researcher said they considered the participant the expert in their own life and wanted to learn what their life was like for them. The participant was initially asked to describe their health condition in their own words. Following this, participants outlined their current school situation, including school year and subjects they enjoyed. After these preliminary conversations, participants were asked to share their preparation activity, which was used as the structure for the remainder of the interview. Participants selected an element of the activity to begin with and the interviewer followed the participant’s lead by asking follow up questions to elicit further detail, examples or reflections. The content of the activity was worked through until the participant was happy they had discussed all they wanted to. The interviews concluded with two final questions asking them for their wish list for changes their school could make (Fig. 6) and their advice for other young people in a similar position (Fig. 7). No topic guide was used for participants who had completed a preparation activity.

For those who did not complete an activity we endeavoured to maintain the participant-led dynamic within the interviews by asking open questions and then following whatever topics the participant chose to raise. We chose questions based on what the participant had already told us about their condition or their school life in the opening part of the interview. For example, questions such as: *“so you mentioned people at school can eat whatever they want to and you have to be a little bit more careful. What’s that like for you?”*, or *“so you said you have just finished year 7…how did the school help you with your health condition in the first year of high school?*

One of the challenges of conducting focus groups with young people is maintaining their concentration and interest in the topic, and using an activity or exercise is recommended [39, 40]. To facilitate our focus groups, participants were asked to focus on the target activity (Fig. 2). In keeping with our approach to the interviews, they were asked to complete this individually beforehand, so they would feel prepared for the group. During the group they then completed this activity together on a shared virtual whiteboard (Jamboard) and discussed any differences they had in the importance of elements of school life.

**Fig. 8:**
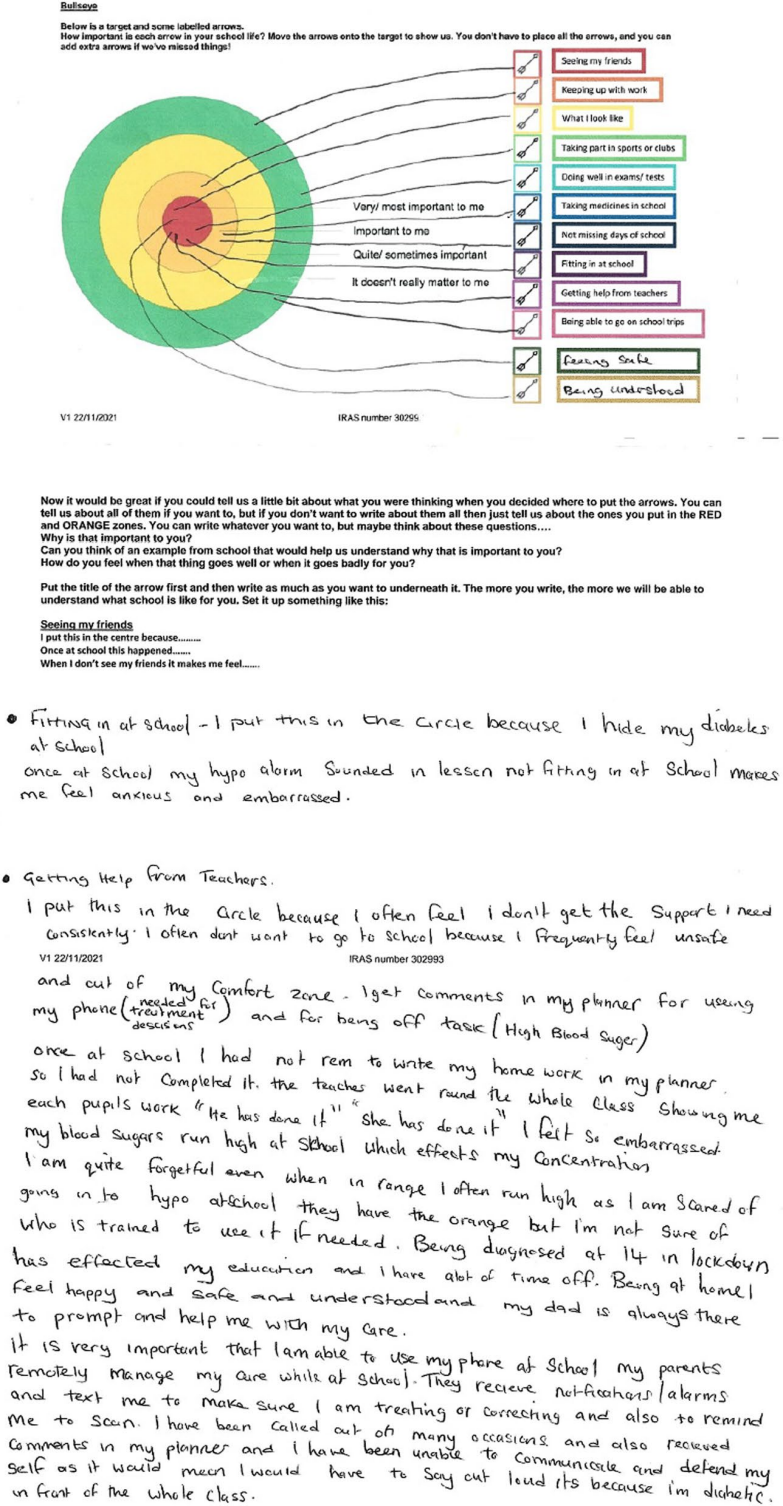
Adapted written task completed by 14 year old from the diabetes clinic

In addition, during the data collection phase researchers adapted the target activity into a written task for several participants who wanted to participate, but did not wish to speak in an interview or focus group (Fig. 8). Their written contributions were then analysed alongside the transcripts of the interviews and focus groups.

The concept and design of preparation activities was arguably the most successful and impactful element of the PPIE and participatory methods combining in this project. The PPIE advisors co-designed activities that appealed to a range of young people and further refined these through the pilot interviews. During data collection the majority of participants completed an activity (54/89). The most popular was the target (42/89), then the Venn diagram (4/89), mood board (4/89) and letter (4/89). Two participants also did their own preparation by making notes of topics they wanted to discuss or a table of topics in order of importance. Having all activities selected by multiple participants showed the range of activities were valuable. When conducting the data collection, both researchers and participants found using the activities as the stimulus for conversation helped the conversation to flow and remain grounded in the young person’s experience.

“*I was able to think of a lot of things that I wouldn’t have thought of just off the top of my head*.”

“*You kind of put the main ideas on there and then you can branch off into other things, but it kind of makes sure, it makes sure that you don’t miss anything. When you kind of talk about it, you realise that it all kind of connects*.”

These reflections show the methods could be empowering for participants and removed some of the pressure to think of answers and examples in the moment. In this way, the approach contributed to an increased power sharing, whereby young people were not left ‘on the back foot’ in research but instead arrived prepared to be an active part of the discourse. In combination with being able to suggest their own question to start the interview, this approach gave them an opportunity to have a sense of control over what was going to be talked about and enhanced the informed consent process.

The researchers reflected on the PPIE advisors’ suggestion of the need for flexibility and choices to be offered in data collection. This proved to be a needed and valuable addition to the methods. Participants predominantly opted for interviews (80/89), with some choosing focus groups (7/89) or the written alternative (2/89). Presenting choices for how to participate was a good initial step towards informed consent, participant driven data collection and increased empowerment of participants. We were also able to include nine participants who said they would not have taken part if the only option was a one-to-one interview. Such feedback underscores the importance of choice, and therefore some element of control, for research participants. Data collection was most commonly scheduled 3pm-6pm on weekdays to fit with after school timings, but other data collection occurred in the evenings or at weekends to suit individual needs. The online nature of this project improved our ability to flexibly accommodate the logistical needs of participants. However, whilst the online interviews were experienced well on both sides, the researchers reflected that the online nature of the focus groups made it more challenging to generate and sustain dialogue between research participants.

### Participatory analysis

Analysis is a stage where young people are rarely included in the research process. To engage with PPIE advisors in the analysis stage of the project, three online group meetings were designed to map onto the stages of the planned approach of thematic analysis [41]:

- Meeting one: familiarisation and coding.
- Meeting two: searching for themes, reviewing themes, and naming themes;
- Meeting three: writing up.

Following a meeting with LYF to discuss the idea and plans for the participatory analysis, five young people volunteered to be PPIE advisors in the analysis stage. In keeping with the preparation activities used in the data collection phase, to help PPIE advisors feel prepared and comfortable in what they were being asked to do they were emailed five anonymised extracts from interview transcripts prior to the first meeting. These had been selected by the research team to represent diversity of participants and content.

**Fig. 9:**
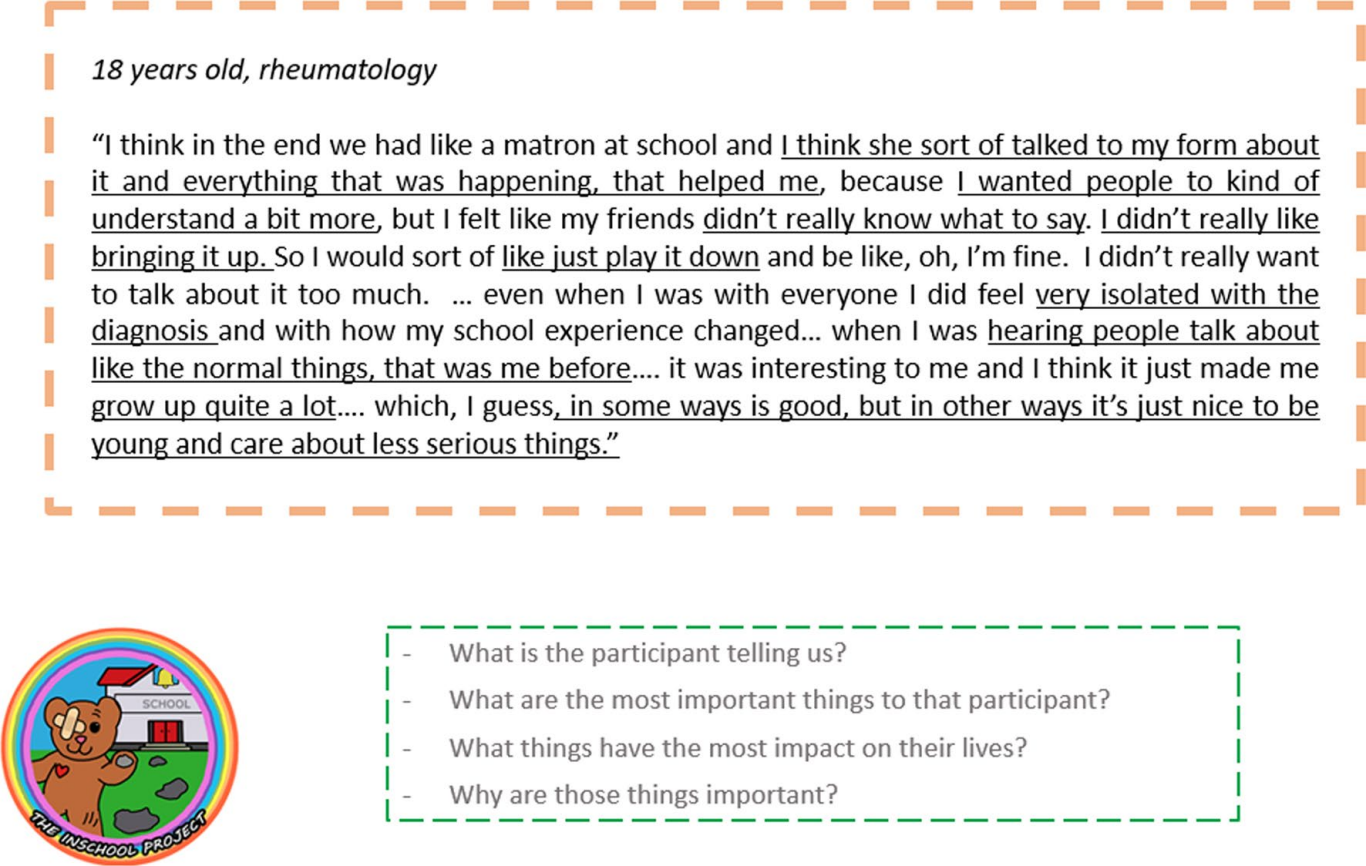
A transcript extract underlined by participatory analysis members

In the first meeting, PPIE advisors were given an introduction to the broad idea of thematic analysis and why the first stage is familiarisation and consideration of individual transcripts. They then discussed the extracts as a group, focussing on what they felt the participant was trying to say and what they thought the important topics were. As a group they underlined key words and phrases (Fig. 9) and discussed why they had made these choices. This was followed by a discussion of the five extracts as a whole, including what was expected or unexpected, how transcripts might be compared, and ways in which the content related to the PPIE advisors’ own experiences.

**Fig. 10:**
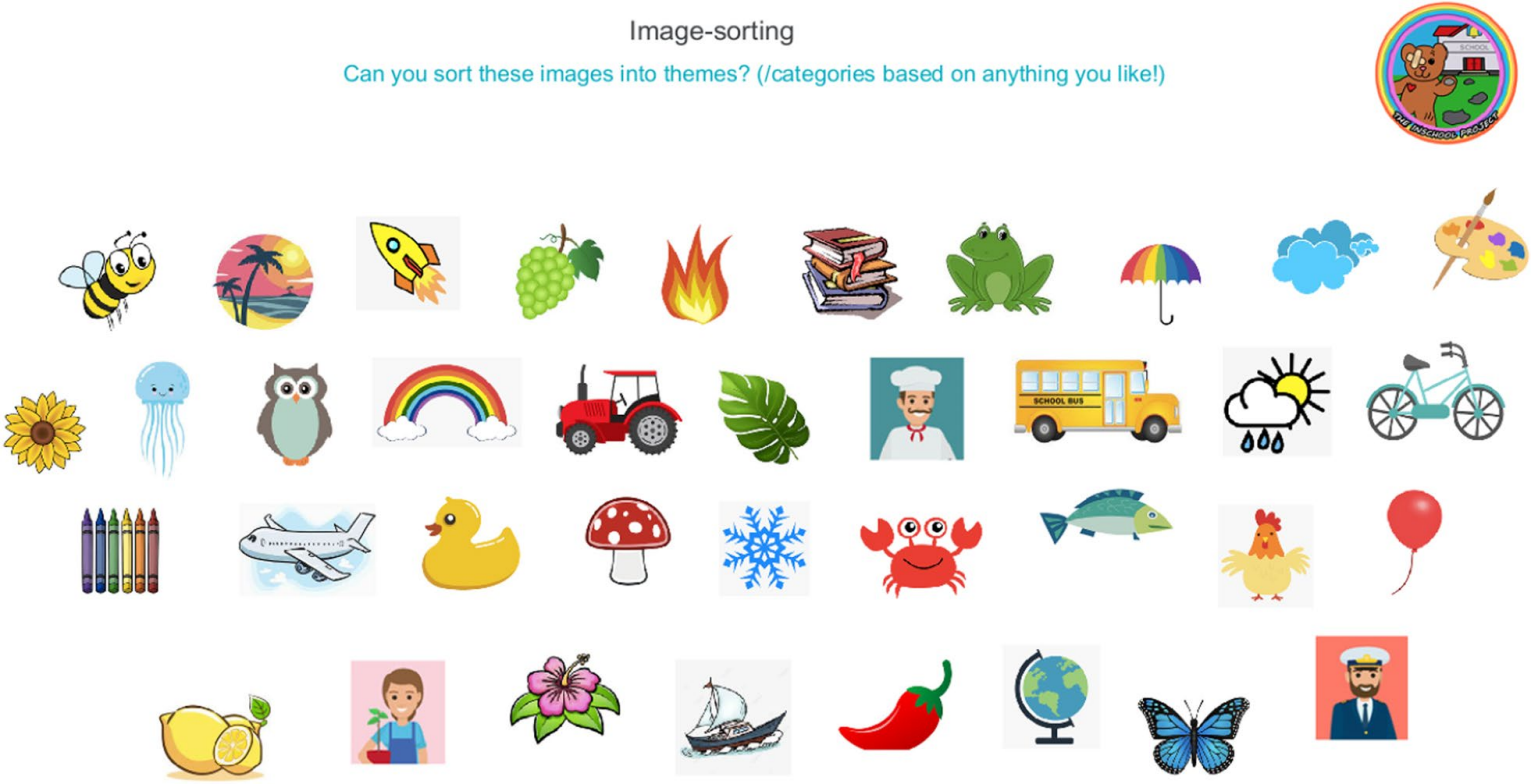
Participatory analysis, image-sorting exercise

In the second meeting PPIE advisors began coding and developing themes, done in three main stages carried out using an interactive whiteboard (Jamboard) that allowed each participant to move images and add labels to the shared screen. Firstly, the advisors took part in an image-sorting exercise, grouping and regrouping images based on anything they saw as shared characteristics (Fig. 10). This was designed to orientate them to the general idea of being able to organise data in a variety of different ways. The PPIE advisors grouped the images variously based on colour, transport, nature, food, and weather. They discussed and reflected on the idea that they each saw these images slightly differently and how none of the categories they created fitted the images perfectly, so therefore there were no right or wrong answers when doing this task.

**Fig. 11:**
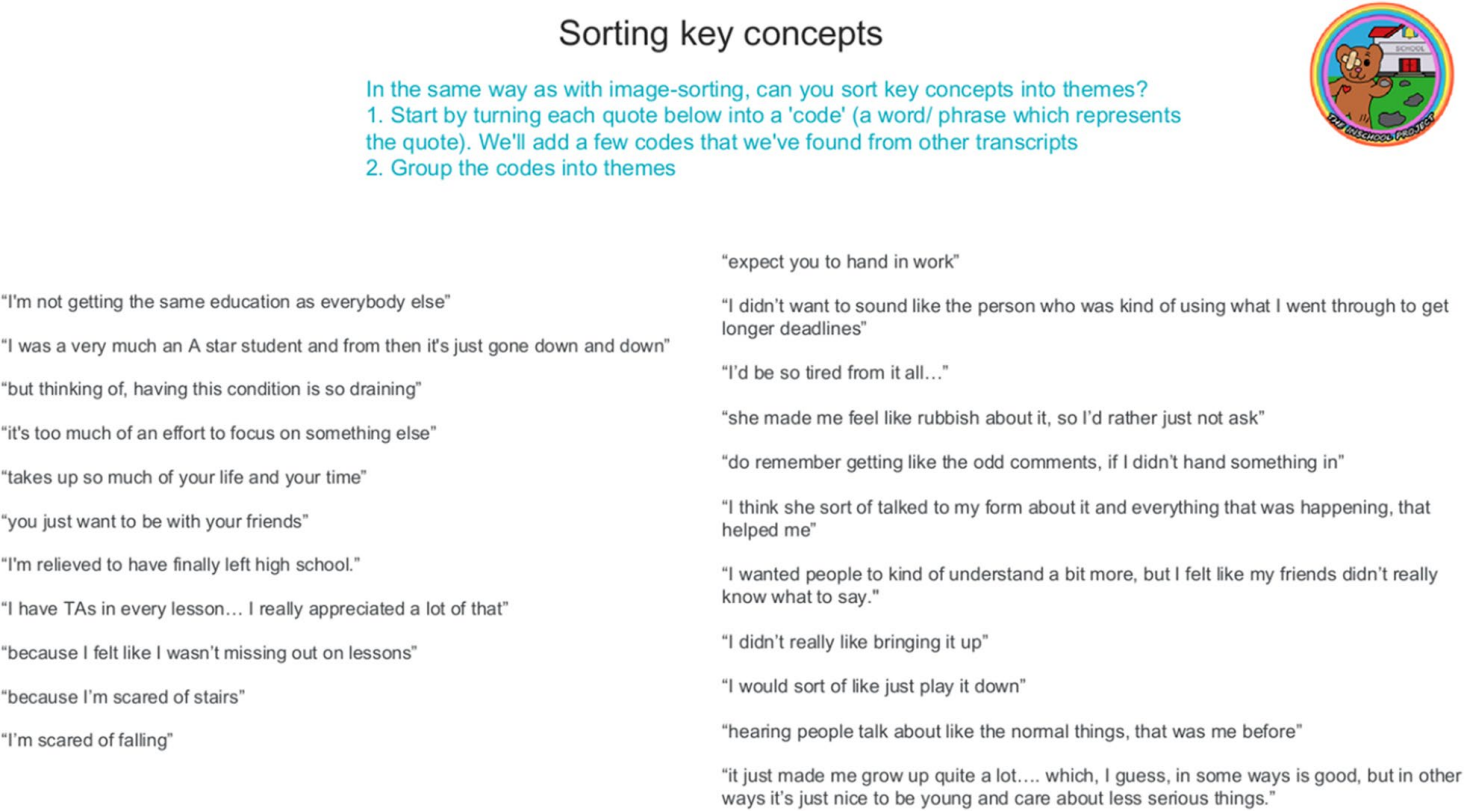
Participatory analysis, code development

Secondly, they took the ideas from the image sorting task and applied them to all of the underlined phrases from the transcripts discussed in the first meeting. Initially they discussed and created one or two word labels or codes for each extract (Fig. 11).

**Fig. 12:**
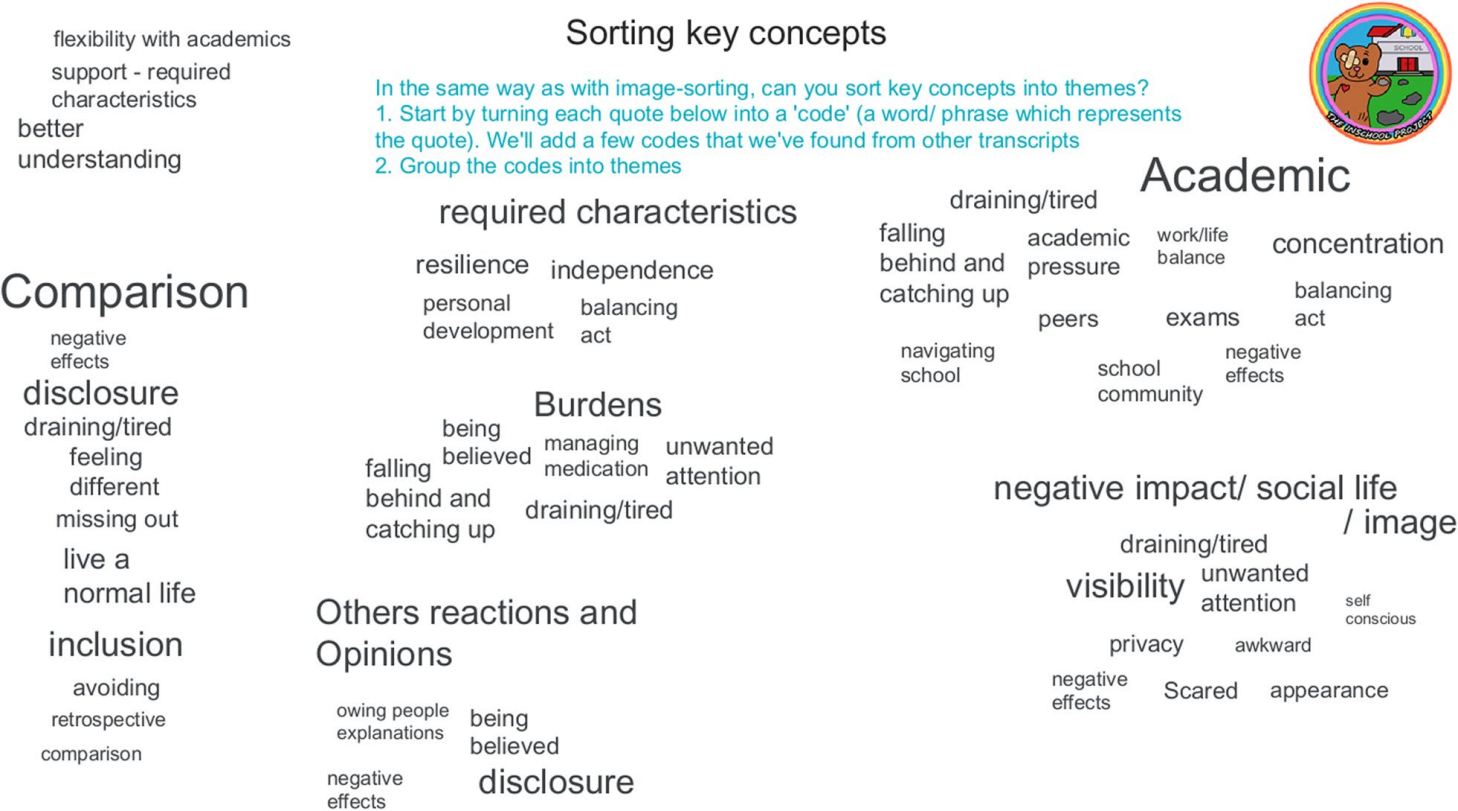
Participatory analysis, theme development

The research team then added some additional codes identified in other transcripts to represent the broader context of the full dataset. PPIE advisors then worked collaboratively to group these codes into themes. They discussed and reworked clusters of codes until there was consensus with the themes they had created (Fig. 12).

Before the final meeting the research team distributed a short video summarising the current analysis output of the qualitative project so that PPIE advisors could have time to reflect and think of questions or comments. In the meeting, having recapped the results, the young people had an informal discussion, offering initial thoughts and questions, highlighting what was expected or unexpected and what they related to their own experiences. They then made suggestions relating to the phrasing and word choice of the results, suggesting ways to clarify or adapt them. To conclude the sessions, the young people shared their thoughts about having taken part and completed a survey to feedback their experience of being a PPIE advisor.

When asked what was challenging about the process, PPIE advisors said that talking in front of people and bringing up personal topics could be difficult in a group context. One member raised that it was challenging but also engaging to be asked to reflect on information and think about conveying their own perspective:

“*It was very thought provoking, but in a good way*.”

They highlighted the most enjoyable aspects of being a PPIE advisor were hearing the opinions of other PPIE advisors and the experiences of the participants seen through the anonymous interview excerpts. They valued being able to offer their own perspectives and experiences to those of other PPIE advisors and the study participants, and seeing how the combination of these voices had the potential to contribute to positive change.

“*I enjoyed taking part in the interview and data collection. I found the data analysis really interesting and informative. I felt like I was helping make change*!”

The participatory analysis meetings produced rich and detailed conversations that built from one meeting to the next. Because we had a stable group who had already supported the project in earlier stages, including two of the pilot interviews, we were able to build trust and rapport which facilitated open discussions. In the periods of time following each meeting the research team reflected on these discussions, looked back at what the PPIE members had produced and the language they had used, which formed an essential and ongoing part of the analysis of the full dataset. PPIE feedback shaped the structure of the themes, how these themes were labelled and conceptualised, and language used to describe each one. Reflecting the language used and emphasis placed on each theme continued into the dissemination of the results through presentations and the final paper [in development].

When asked if they had learnt anything from the analysis sessions, PPIE advisors responded they had a better awareness of the commonalities in experiences of a range of different health conditions, that changes needed to be and could be made, and that they were not alone in their own experiences.

“*Even though a range of people with a variety of health conditions took part in the interviews, there were many overlaps with experiences and needs. This shows that action needs to be taken. I learnt that I am not alone in my school experiences*.”

### Dissemination

To conclude their involvement in the INSCHOOL project one of our PPIE advisors, who was involved in all stages outline above, co-presented at a regional seminar. The seminar was for professionals interested in developing projects using participatory methods with young people. Our advisor contributed to every stage of the presentation by providing her perspective and experience of each stage of her involvement in the research. She also helped respond to questions from the audience and co-facilitated an activity session where attendees completed and discussed one of the preparation activities. Our PPIE advisor really valued the opportunity to join our presentation team at the regional seminar. She said she *“enjoyed being included in the seminar and it was an amazing experience”*.

## Discussion

This paper has provided examples of PPIE work with young people and how this contributed to the design of participant-driven methods in the INSCHOOL project. The INSCHOOL project adopted a pragmatic approach to facilitate young people’s voices as advisors, co-designers and research participants, rather than aligning to a specific theoretical or methodological discipline [7]. A pragmatic approach allowed the research team and PPIE advisors to develop, and flexibly adapt, activities and methods to fit the research topic and the needs of the young people involved. The LYF and PPIE advisors were involved in, and consulted about, the INSCHOOL project from the outset, and were then given opportunities to be more actively involved in supporting stages of the research process through pilot interviews, analysis, and dissemination, which is less common in research [6].

Time and resource for the PPIE aspects of the INSCHOOL project were planned and costed into the initial application, which addressed concerns of this as a barrier [10, 12, 18] and reflects previous successful examples of planning for PPIE work [14]. Having this work as a specific aim of the project that was included throughout the timeline gave the research team the permission and impetus to focus on PPIE as equal in value to the data collection, rather than an additional time intensive activity.

Power imbalances hamper knowledge generation and limit the learning we can gain from young people. To begin to address power imbalances, research has looked to develop methodologies allowing young people to express themselves in ways that matter to them, but also involve young people in the design of research [42]. Adopting creative methods to encourage a participant-driven approach can begin to address power imbalances through a level of control over the process [43], as well as improving participant engagement by facilitating freedom of expression [44]. The INSCHOOL project attempted to address power imbalances throughout the entire research process: PPIE at the inception of the project allowed young people to shape the initial question; thorough multi-stage informed consent, selection of an opening question and the creative preparation activities enabled participants to feel comfortable and have increased control of the process before data collection began; using the preparation activities as the stimulus for the interviews and focus groups, and positioning them as the experts in their condition empowered young people to be able to lead more aspects of the data collection itself; PPIE advisors shaped the analysis of the data; and one advisor influenced how this work was discussed in dissemination.

When considered on the continuum of participatory research [19], the INSCHOOL project achieved a high level of engagement with young people. However, there were barriers to achieving a full egalitarian research partnership in every phase because of our difficulties in involving young people directly in data collection. Although other research projects have achieved this [14], the potentially sensitive nature of the research topic made it challenging for researchers and PPIE advisors to devise an appropriate method for young people to conduct the qualitative data collection whilst adhering to consent and the safety of all involved. Putting young people in a position where sensitive topics may be raised and they would have to manage the subsequent discussion was felt to be too challenging to prepare them for, but was an essential aspect of the information aimed for within the research. However, PPIE advisors significantly contributed to the design of the data collection methods, so were still able contribute indirectly.

The INSCHOOL project has been greatly enhanced because of the PPIE and participatory methods. Young people encouraged and co-designed a flexible and participant centred approach, and then monitored the project through regular PPIE. At every stage of the project they were able to steer the research team back towards the experiences, language and perspectives of young people. Without their regular involvement and advice the essential grounding in the experiences of young people would have been difficult for the research team to confidently achieve.

## Conclusions

Young people have much knowledge and guidance to offer researchers and they themselves value opportunities to share their experiences and insights. The INSCHOOL project has taken the position that young people are research partners who can help elevate the quality and meaningfulness of research about them and for them. We have shown that researchers can meaningfully involve young people in the research process, not just as ad hoc consultants Co-designed participant-driven research methods have facilitated the INSCHOOL project to address power imbalances in research and prioritise the voices of young people. The flexible and creative pre-interview methods developed in collaboration with young people are a particular way to move towards more age-appropriate forms of research. Engaging with young people across the lifetime of the project has added value at every stage by keeping the project grounded in the experiences of those that the research aims to benefit and by offering insightful alternative perspectives on methods and findings.

## Data Availability

The datasets referred to during the current paper are available from the corresponding author on reasonable request. The final results of this research will be available in a future publication.
